# Dimension control of in situ fabricated CsPbClBr_2_ nanocrystal films toward efficient blue light-emitting diodes

**DOI:** 10.1038/s41467-020-20163-7

**Published:** 2020-12-22

**Authors:** Chenhui Wang, Dengbao Han, Junhui Wang, Yingguo Yang, Xinyue Liu, Sheng Huang, Xin Zhang, Shuai Chang, Kaifeng Wu, Haizheng Zhong

**Affiliations:** 1 MIIT Key Laboratory for Low-Dimensional Quantum Structure and Devices, School of Materials Science & Engineering, Beijing Institute of Technology, 100081 Beijing, China; 2 State Key Laboratory of Molecular Reaction Dynamics and Collaborative Innovation Center of Chemistry for Energy Materials (iChEM), Dalian Institute of Chemical Physics, Chinese Academy of Sciences, 116023 Dalian, China; 3 Shanghai Synchrotron Radiation Facility (SSRF), Shanghai Institute of Applied Physics, Chinese Academy of Sciences, 201204 Shanghai, China

**Keywords:** Materials for devices, Nanoscale materials

## Abstract

In the field of perovskite light-emitting diodes (PeLEDs), the performance of blue emissive electroluminescence devices lags behind the other counterparts due to the lack of fabrication methodology. Herein, we demonstrate the in situ fabrication of CsPbClBr_2_ nanocrystal films by using mixed ligands of 2-phenylethanamine bromide (PEABr) and 3,3-diphenylpropylamine bromide (DPPABr). PEABr dominates the formation of quasi-two-dimensional perovskites with small-*n* domains, while DPPABr induces the formation of large-*n* domains. Strong blue emission at 470 nm with a photoluminescence quantum yield up to 60% was obtained by mixing the two ligands due to the formation of a narrower quantum-well width distribution. Based on such films, efficient blue PeLEDs with a maximum external quantum efficiency of 8.8% were achieved at 473 nm. Furthermore, we illustrate that the use of dual-ligand with respective tendency of forming small-*n* and large-*n* domains is a versatile strategy to achieve narrow quantum-well width distribution for photoluminescence enhancement.

## Abstract (web summary)

Designing efficient blue perovskite LEDs by using mixed halides perovskite is still a challenge, limited mainly by the phase segregation issue. Here, the authors demonstrate in situ fabrication of quasi-2D CsPbClBr2 nanocrystal films with mixed ligands to overcome the constraint.

## Introduction

Perovskite light-emitting diodes (PeLEDs) are emerging as an alternative display technology due to their high color purity, high external quantum efficiency (EQE), and solution processability^1–8^. Benefiting from the ionic feature of the metal halide perovskite, PeLEDs can be directly fabricated through in situ fabrication technique by spin-coating perovskite precursor solution on targeting substrates^9–14^. Since the first report of room-temperature-operated perovskite-based electroluminescence (EL) devices in 2014^1^, the maximum EQEs of green, red, and near-infrared PeLEDs have exceeded 20%, which are comparable to organic light-emitting diodes and quantum dot light-emitting diodes^15–22^. However, the performance of blue PeLEDs still lags behind their green, red, and near-infrared counterparts, especially in the pure blue region (455–475 nm) toward display applications, which is an obstacle to develop full-color display technology^23,24^.

Basically, the spectral modulation of perovskite light emitters can be achieved by tuning the composition, size, and/or dimension^25–29^. Blue emissive three-dimensional (3D) perovskite nanocrystals have been successfully fabricated by reducing the size of bulk perovskites or introducing mixed halides^30–33^. However, the blue EL devices based on such small-sized perovskite nanocrystals suffer from the efficiency and stability issues mainly due to the complicated purification and phase separation^31,34^. An alternative strategy for achieving efficient blue PeLEDs is the construction of quasi-two-dimensional (quasi-2D) perovskites with multiple quantum wells^35–38^. The photoluminescence (PL) properties of these quasi-2D perovskites are strongly correlated with the energy transfer from small-*n* to large-*n* domains^39–43^. It was found that a flattened quantum-well width distribution (QWD) of quasi-2D perovskite is essential to promote carrier transport and reduce extra energy loss for achieving high-performance photovoltaic devices^44,45^. However, the influence of QWD on EL devices has been less investigated.

It has been learned that the QWD can be controlled by adjusting the ratio of the precursor mixture, or ligand engineering^35,44–46^. Herein, we demonstrate that the use of dual ligand is an effective strategy for controlling the QWD of in situ fabricated CsPbClBr_2_ nanocrystal films. 2-Phenylethanamine bromide (PEABr) has been intensively investigated as an effective ligand to form small-*n* domains, while 3,3-diphenylpropylamine bromide (DPPABr) is useful in forming large-*n* values. Judiciously selecting the ratio of the two ligands narrows down the QWD with central domination of *n* = 4. Such effective dimension control facilitates efficient energy transfer, resulting in strong blue emission at the wavelength of 470 nm with a PL quantum yield (PLQY) up to 60%. The use of dual ligand with respective tendency of forming small-*n* and large-*n* domains is a versatile strategy to achieve narrow QWD for enhancing the PL properties. Based on the optimized films obtained from a mixture of PEABr and DPPABr, efficient blue-emitting EL devices with a maximum EQE of 8.8% were achieved at the wavelength of 473 nm.

## Results

### Fabrication and structural analysis of CsPbClBr_2_ nanocrystal films

**Fig. 1:**
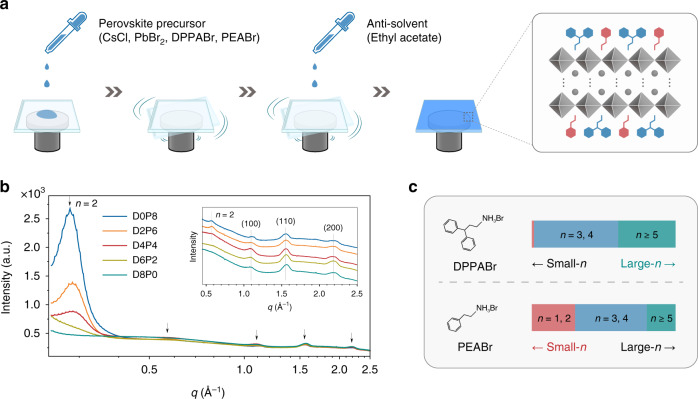
Structural characterizations of the CsPbClBr_2_ nanocrystal films. **a** Schematic illustration of the in situ fabrication process for CsPbClBr_2_ nanocrystal films. **b** The integrated intensity-*q* relations of GIWAXS patterns for the CsPbClBr_2_ nanocrystal films with different ratios between DPPABr and PEABr. Inset: partial enlarged intensity-*q* curves (0.45–2.5 Å^−1^). **c** Schematic diagram of QWD for DPPABr-only and PEABr-only CsPbClBr_2_ nanocrystal films.

Figure 1a schematically shows the in situ fabrication process of blue-emitting CsPbClBr_2_ nanocrystal films. In order to control the QWD, a dual-ligand strategy is adapted by adding organic ligands DPPABr and PEABr into the precursor solutions with various ratios. The molar ratio of CsCl, PbBr_2_, and organic ligands (DPPABr and PEABr) is set as 1.1:1:0.1(*x* + *y*), where *x* and *y* are integers that represent the quantities of DPPABr and PEABr in the precursor (*x* + *y* = 8). In the following, the resulting CsPbClBr_2_ films are labeled as “D*x*P*y*” to differ the precursor solutions. The slight excess of CsCl in the precursor ensures the formation of pure CsPbClBr_2_ phase^47^. During the spin coating process, ethyl acetate, used as anti-solvent, is quickly dropped before color change to induce rapid nucleation and partly remove excess organic ligands^5,11,37^. Supplementary Movie 1 shows the formation process and the corresponding sample of D4P4.

The as-fabricated CsPbClBr_2_ nanocrystal films were characterized by applying grazing incidence wide-angle X-ray scattering (GIWAXS) analyses (see Supplementary Fig. 1 for the GIWAXS images). Figure 1b shows the integrated GIWAXS data of the films fabricated by using different molar ratios of the two ligands. All samples exhibit three peaks at 1.09, 1.55, and 2.18 Å^−1^, which can be attributed to the (100), (110), and (200) crystal planes of cubic 3D CsPbClBr_2_ nanocrystals (Supplementary Fig. 2 and Supplementary Table 1). Furthermore, two distinctive peaks at 0.28 and 0.57 Å^−1^ can be observed for the sample of D0P8, corresponding to the diffraction and secondary diffraction of the *n* = 2 perovskites^37,48^. The diffraction peaks of the laminar structure gradually become weaken for the samples of D2P6 and D4P4 with the DPPABr/PEABr ratio increasing and disappear for the samples of D6P2 and D8P0. The evolution of the diffraction peaks indicates that large-*n* domains become dominated for the samples obtained with a higher DPPABr content. As schematically described in Fig. 1c, the use of DPPABr generally induces the formation of quasi-2D perovskites with large-*n* domains, while PEABr dominates the formation of small-*n* domains. It is deduced that the *n* values of the resulting films can be tuned by varying the ratio between DPPABr and PEABr, providing a practical methodology for controlling the QWD.

### Optical properties of the CsPbClBr_2_ nanocrystal films

**Fig. 2:**
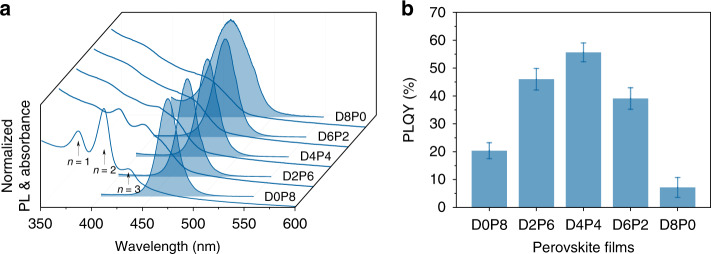
Optical measurements of the CsPbClBr_2_ nanocrystal films. **a** Steady-state PL and absorption spectra and **b** PLQYs of the CsPbClBr_2_ nanocrystal films obtained with different ratios between DPPABr and PEABr. The error bars correspond to the standard deviation.

We further performed optical measurements of CsPbClBr_2_ nanocrystal films with different ratios of the mixed ligands. As shown in Fig. 2a, excitonic absorption peaks can be clearly identified for the samples derived with a higher ratio of PEABr, which appears at ~386, ~412, and ~435 nm, corresponding to the quantum-well domains with *n* values of 1, 2, and 3, respectively. With DPPABr increasing, the excitonic peaks of the resulting quasi-2D perovskite films become less identifiable, implying a reduction of the small-*n* domains. The samples show blue PL emissions with emission peak at 466, 467, 470, 471, and 458 nm and full width at half maximum (FWHM) of 22.2, 21.7, 22.6, 25.4, and 45.4 nm for the samples of D0P8, D2P6, D4P4, D6P2, and D8P0, respectively. It is noted that the sample of D8P0 shows a broader PL spectrum, implying the existence of multiple emissions. As shown in Fig. 2b, the PLQYs of these samples are correlated with the ratio of the two ligands. A maximum PLQY of 60% is obtained for the sample of D4P4.

To illustrate the versatility of the dual-ligand strategy for PL enhancement, we further investigated other ligands of similar types with PEABr or DPPABr, 3-phenylpropylamine bromide (PPABr), 4-phenylbutylammonium bromide (PBABr), n-butylammonium bromide (BABr), iso-butylammonium bromide (iBABr), 2,2-diphenylethanamine bromide (DPEABr), and 1-(1-naphthyl)ethylamine bromide (NEABr). The absorption spectra of these resulting CsPbClBr_2_ films with single ligand are summarized in Supplementary Fig. 3. The as-fabricated films using PPABr, PBABr, BABr, and iBABr show significant absorption peaks of the small-*n* domains (*n* = 1, 2, 3), which are similar with that of PEABr. However, these peaks become less identifiable in the films obtained from DPEABr and NEABr, exhibiting the absorption feature of large-*n* domains (*n* > 3), which are similar with that of DPPABr. Importantly, the mixing of these two different types of ligands (molar ratio of 1:1) can tune the domain feature of the resulting films to a moderate *n* value (Supplementary Fig. 4), resulting in PL enhancement (Supplementary Fig. 5). These results imply that the use of dual ligand is a versatile strategy to control the QWD in quasi-2D perovskite nanocrystal films.

### Transient absorption (TA) spectroscopic analysis

**Fig. 3:**
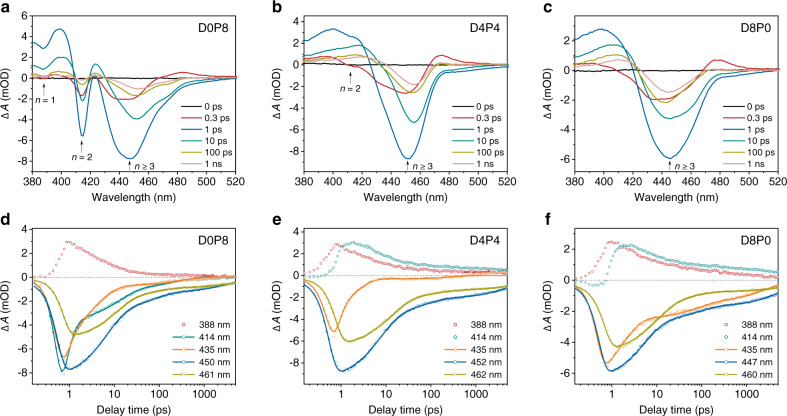
TA characterizations of the CsPbClBr_2_ nanocrystal films. **a**–**c** TA spectra at different delay times for the samples of **a** D0P8, **b** D4P4, and **c** D8P0, respectively. **d**–**f** TA kinetics of *n* = 1, 2, 3, 4 and *n* ≥ 5 domains for the samples of **d** D0P8, **e** D4P4, and **f** D8P0, respectively. Solid lines are the fitting curves using exponential function.

TA measurements were carried out to derive the correlations between QWD and PL properties of the resulting CsPbClBr_2_ nanocrystal films (see Supplementary Fig. 6 for the TA color maps). Figure 3a–c shows the TA spectra at different delay times for the samples of D0P8, D4P4, and D8P0. Two distinctive ground-state bleach peaks at ~388 and ~414 nm can be observed for the sample of D0P8, corresponding to *n* = 1 and *n* = 2 domains, which are consistent with the observed excitonic peaks of small-*n* domains in the steady-state absorption spectra (Fig. 2a). However, the bleach peak of *n* = 1 is absent for the samples of D4P4 and D8P0, suggesting a lower content of *n* = 1 domains in these two films. In addition, the sample of D4P4 shows a bleach peak at ~412 nm at the first 300 fs after excitation, which is not observed for the sample of D8P0. It suggests that the *n* = 2 domains are reduced for the sample with higher content of DPPABr, which agrees with the GIWAXS analysis. We further analyzed the TA kinetics of the CsPbClBr_2_ nanocrystal films, as shown in Fig. 3d–f. The TA kinetic of D0P8 shows one positive feature of the *n* = 1 domain, which can be explained that the bleach signal is overlapped by the strong excited-state absorption signal of *n* = 2 domains^48^. Similarly, positive features are observed in the *n* = 1 and *n* = 2 kinetics for the samples of D4P4 and D8P0, as the bleach signals are overlapped by the excited-state absorption signal of next-large *n* value (*n* = 2 and 3) domains. Based on the discussions above, the strong positive features of TA kinetics for the samples of D4P4 and D8P0 can be attributed to the lower content of *n* = 1 and *n* = 2 domains. Thus it can be concluded that the formation of small-*n* domains (*n* = 1, 2) is suppressed as the proportion of DPPABr increases.

On the other hand, all the samples show an inhomogeneous broad bleach peak at 425–470 nm (Fig. 3a–c). Note that at least two peaks are packed in the broad peak at the first 300 fs after excitation in all TA spectra. The broad peaks can be attributed to the distribution of a series of quantum-well domains (*n* ≥ 3)^48,49^. Combined with the steady-state absorption spectra (Fig. 2a), three bleach peaks at ~435, ~450, and ~461 nm can be attributed to the quantum-well domains of *n* = 3, *n* = 4, and *n* ≥ 5, respectively. The domains of *n* ≥ 5 cannot be distinguished due to the small gradient distribution of their bandgap energy^48^. The TA kinetics (Fig. 3d–f) show that the maxima of each bleach peaks are delayed successively, revealing the continuous energy transfer from small-*n* to large-*n* domains and the distribution of multiple quantum wells^50^.

### Carrier dynamic analysis

**Fig. 4:**
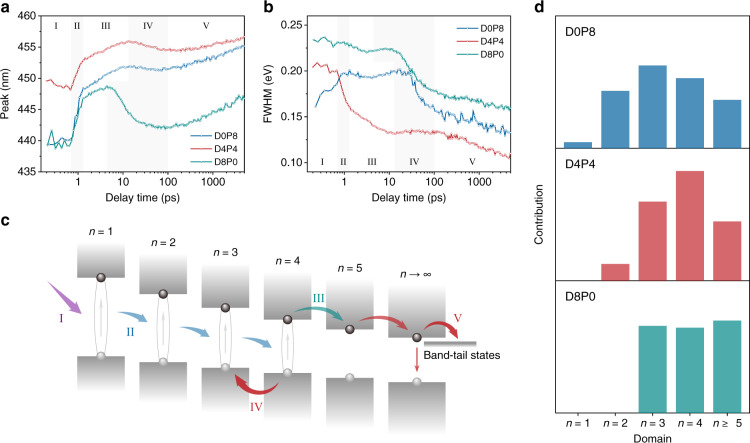
The effects of QWD on their carrier dynamics. **a**, **b** Extracted **a** peak and **b** FWHM evolution from the broad bleach peak (425–470 nm) for the samples of D0P8, D4P4, and D8P0. **c** Schematic diagram of carrier behaviors after excitation. The carrier recombination progress can be divided into five stages: I, carrier formation; II, exciton transfer; III, charge transfer; IV, reverse charge transfer; V, continuous charge transfer and recombination. **d** Contribution of each domain in the CsPbClBr_2_ nanocrystal films as estimated from the TA spectra.

Based on the above results, we further analyzed the effects of QWDs on their carrier dynamics. As shown in Fig. 3a–c, compared with D0P8 and D8P0, D4P4 shows a narrower FWHM of the broad bleach peak (D4P4, 0.13 eV; D0P8, 0.20 eV; D8P0, 0.22 eV; at ~10 ps), implying the narrowed QWD. Figure 4a, b show the time evolution of the peak wavelength and FWHM extracted from the broad bleach peak (425–470 nm). According to the previous discussions and the TA data, the carrier dynamics can be divided into five stages upon excitation: I, carrier formation; II, exciton transfer; III, charge transfer; IV, reverse charge transfer; V, continuous charge transfer and recombination, as shown in Fig. 4c. In stage I, photo-induced carriers are formed within the first several hundred femtoseconds after photo-excitation, which can be reflected by the increasing bleach peak signal. The evolutions of the peak and FWHM illustrate the continuous competition of bleach signals from different *n* value domains. In stage II, an ultrafast exciton transfer from small-*n* domains (*n* ≤ 3) to large-*n* domains (*n* ≥ 4) occurs in the time span of 0.7–1.3 ps^39,40,50,51^, resulting in a fast redshift of the broadened bleach peak. In stage III, excitons begin to dissociate into free charge carriers and charge transfer occurs subsequently within a few picoseconds^51–55^, which induces a further redshift of the bleach peak. At the end of stage III, carriers mainly concentrate on the *n* = 4 domains. For the sample of D4P4, the rapid decrease of FWHM of the bleach peak represents the efficient energy transfer process from small-*n* domains to large-*n* domains. In contrast, the decrease of bleach peak FWHM for the samples of D0P8 and D8P0 is less obvious, which can be ascribed to the increasing bleach signal of larger *n* domains (*n* ≥ 5) at ~10 ps, as shown in Fig. 3a, c and Supplementary Fig. 7, implying a wider QWD than that of D4P4.

In stage IV, the bleach peak of the samples exhibits a blueshift in the following ~100 ps after stage III, which can be assigned to interwell reverse charge transfer from large-*n* domains to small-*n* domains, especially from *n* = 4 to *n* = 3^51,52^. We highlight that the reverse charge transfer is detrimental for achieving high PLQYs, as energy cannot concentrate on the effective emission domains. The most obvious blueshift (~7 nm) of the bleach peak is observed for the sample of D8P0, indicating the most severe reverse transfer. This explanation is also supported by the observed PL features of broad PL spectrum and low PLQY of D8P0 (Fig. 2a, b). In stage V, charge carriers continuously transfer to large-*n* domains and/or band-tail states and recombine radiatively or non-radiatively. This process is evident by continuous redshift as well as the decrease of FWHM of the bleach peak. Compared with the sample of D4P4, the bleach peaks of D8P0 and D0P8 exhibit a sharper redshift, implying a broader *n* value distribution and a larger proportion of band-tail states in these samples. Since the band-tail states usually correlate with defect states^56,57^, the transition from large-*n* domains to band-tail states in D8P0 and D0P8 may account for their relatively low PLQYs.

We further estimated the contribution of each domain by adapting the reported method^50^, as shown in Fig. 4d. A higher proportion of small-*n* (*n* = 1, 2) domains is obtained in PEABr-only films, which may lead to the non-radiative recombination and inefficient energy transfer and therefore result in a low-efficiency emission^58^. The formation of such small-*n* domains is inhibited in DPPABr-only films. However, the disordered energy states due to the broad QWD as mentioned above lead to significant reverse charge transfer and band-tail states as well as a low PL efficiency. Eventually, the mixed use of the two ligands reduces the content of small-*n* domains while narrows the QWD to concentrate on *n* = 4 domain for realizing effective energy and charge transfer to the recombination center, which results in a high-efficiency blue emission.

### Device structure and performance

**Fig. 5:**
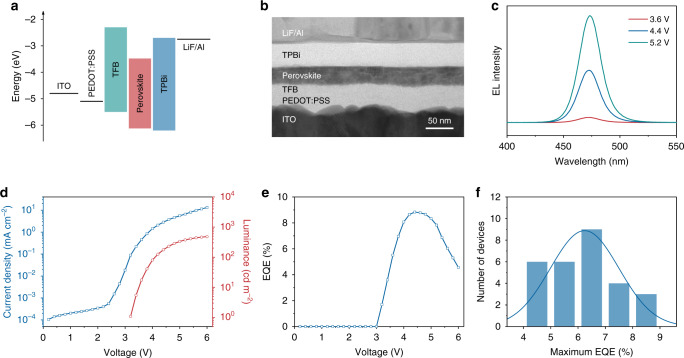
Characteristics of blue PeLEDs. **a** Energy level diagram of the EL devices. **b** Cross-sectional TEM image of the multi-layered EL device. **c** EL spectra under forward biases of 3.6, 4.4, and 5.2 V. **d** Current density–luminance–voltage characteristic of the best-performance device. **e** EQE–voltage characteristic of the best-performance device. **f** Histogram of maximum EQEs from 28 devices.

Based on the optimized CsPbClBr_2_ nanocrystal films (D4P4), we fabricated EL devices using the following structure (Fig. 5a, b): indium tin oxide (ITO, ~150 nm)/poly(3,4-ethylenedioxythiophene):poly(styrenesulfonate) (PEDOT:PSS, ~30 nm)/poly(9,9-dioctylfluorene-co-N-(4-butylphenyl)-diphenylamine) (TFB, ~15 nm)/perovskite (~30 nm)/2,2′,2″-(1,3,5-benzinetriyl)tris(1-phenyl-1H-benzimidazole) (TPBi, ~40 nm)/lithium fluoride (LiF, ∼1 nm)/aluminum (Al, ∼100 nm). As noted in Supplementary Fig. 8, the energy levels of D4P4-based films were determined by applying ultraviolet photoelectron spectroscopy (UPS) measurement. Figure 5c shows the EL spectra under forward bias of 3.6, 4.4, and 5.2 V. The EL peak is located at 473 nm with an FWHM of 22 nm, corresponding to the Commission Internationale de l´Eclairage color coordinates of (0.120, 0.088). Figure 5d shows the current density–luminance–voltage curves of the best-performance blue PeLED. A maximum luminance of 482 cd m^−2^ is achieved at 6.0 V and a maximum EQE of 8.8% is achieved at 4.4 V with a luminance of 177 cd m^−2^ (Fig. 5e). The performance of 28 devices is summarized in Fig. 5f and Supplementary Fig. 9. The statistical data show an average EQE of 6.2% with an average luminance of 442 cd m^−2^. In addition, we also investigated the device performance based on the other CsPbClBr_2_ nanocrystal films for comparison (Supplementary Fig. 10). Maximum EQEs of 2.8% and 0.05% are achieved for D0P8- and D8P0-based devices, respectively. The device stability of D4P4-based PeLED was tested at a constant current density of 1.95 mA cm^−2^ with an initial luminance around 100 cd m^−2^. The *T*_50_ defined as the time when luminance drops to 50% of its initial value is 6.3 min (Supplementary Fig. 11). Although our device achieves a record EQE in the pure blue region (455–475 nm) toward display applications (Supplementary Fig. 12), it still faces problems such as the spectral shift from 473 to 476 nm during the operation (Supplementary Fig. 13) due to the phase separation under high electric field^59^. Further studies are essential to address the source of the instability of halogen-mixed perovskites and apply high-efficiency films to high-performance devices.

## Discussion

In conclusion, we demonstrate the dimension control of in situ fabricated CsPbClBr_2_ nanocrystal films by using mixed ligands of PEABr and DPPABr toward efficient blue PeLEDs with a maximum EQE of 8.8% at the wavelength of 473 nm. PEABr and DPPABr dominate the formation of quasi-2D perovskites with small-*n* domains and large-*n* domains, respectively. The dual-ligand strategy inhibits the formation of small-*n* and large-*n* domains and thus narrows down the QWD with central domination of *n* = 4. The TA analyses illustrate that the narrowed QWD reduces the reverse charge transfer from large-*n* to small-*n* domains and the transition to band-tail states with non-radiative recombination, which results in efficient blue emission. This strategy of dimension control through dual ligand provides an effective method to put forward the development of blue PeLEDs for display applications.

## Methods

### Materials

CsCl (super dry, 99.99%), PbBr_2_ (99.99%), and LiF (99.85%) were purchased from Alfa-Aesar. DPPA (>98%) and DPEA (>98%) were purchased from Aladdin. NEA (>98%) was purchased from Meryer. PEABr (>99.5%), PPABr (>99.5%), PBABr (>99.5%), BABr (>99.5%), iBABr (>99.5%), PEDOT:PSS (Clevios P VP AI 4083), and TFB (>99%) were purchased from Xi'an Polymer Light Technology Corp. Dimethyl sulfoxide (DMSO) (super dry, with molecular sieves, 99.8%), ethyl acetate (super dry, with molecular sieves, 99.9%), and chlorobenzene (super dry, with molecular sieves, 99.9%) were purchased from J&K Chemical Ltd. TPBi (>99%) was purchased from Luminescence Technology Corp. All the materials were used as received.

DPPABr, DPEABr, and NEABr were synthesized by the reaction of DPPA, DPEA, or NEA with HBr^11^. Perovskite precursor solutions for CsPbClBr_2_ nanocrystal films (D*x*P*y*) were prepared by dissolving CsCl, PbBr_2_, DPPABr, and PEABr with a molar ratio of 1.1:1:0.1*x*:0.1*y* (*x* + *y* = 8) in DMSO, at a concentration of 0.2 mol L^−1^ under stirring for 4 h at room temperature.

### Optical and morphological characterization

PL spectra were measured by using a F-380 fluorescence spectrometer (Tianjin Gangdong Science and Technology Development Co., Ltd.). Steady-state ultraviolet–visible (UV-vis) absorption spectra were measured by using a UV-6100 UV-vis spectrophotometer (Shanghai Mapada Instruments Co., Ltd.). The PLQYs of the films were obtained by using a fluorescence spectrometer with an integrated sphere (C9920-02, Hamamatsu Photonics Co., Ltd.) excited at the wavelength of 397 nm from an LED light source. UPS spectrum was captured by a multi-technique analysis system (ESCALAB 250Xi, Thermo Fisher Scientific Inc.). Cross-sectional transmission electron microscope (TEM) measurement was performed by Toray Research Center, Inc. The sample was prepared by using a focused-ion-beam system (Helios NanoLab 660, FEI Ltd.) and characterized by using a H-9000UHR microscope (Hitachi Ltd.) with an accelerating voltage of 200 kV.

### GIWAXS characterization

GIWAXS measurements were performed at beamline BL14B1 of Shanghai Synchrotron Radiation Facility^60^. Wavelength of the X-ray beam was 1.24 Å. The scattering patterns were obtained with a grazing incidence angle of 0.1° and an exposure time of 30 s. The distance between the sample and the detector was 361 mm.

### TA spectroscopy characterization

The femtosecond pump–probe TA measurements were performed using a regenerative amplified Ti:sapphire laser system (Coherent Inc., 800 nm, 70 fs, 6 mJ per pulse and 1 kHz repetition rate) and a femto-TA100 spectrometer (Time-Tech Spectra Ltd.). The 800-nm output pulse from the regenerative amplifier was split into two parts with a 50% beam splitter. The transmitted part was used to pump a TOPAS Optical Parametric Amplifier, which generated a wavelength-tunable laser pulse from 250 nm to 2.5 μm as pump beam. The reflected 800-nm beam was split again into two parts. One part with <10% was attenuated with a neutral density filter and focused into a 2-mm-thick sapphire window to generate a white light continuum to be used for probe beam. The probe beam was focused with an Al parabolic reflector onto the sample. After sampling, the probe beam was collimated and then focused into a fiber-coupled spectrometer with complementary metal–oxide–semiconductor sensors and detected at a frequency of 1 KHz. The pump pulses were chopped by a synchronized chopper at 500 Hz, and the absorbance change was calculated with two adjacent probe pulses (pump blocked and pump unblocked). The wavelength and intensity of the pump pulse were 365 nm and 15 μJ cm^−2^, respectively.

Chirp-corrections were performed on the TA spectra. TA kinetics were fitted to the convolution of the instrument response and a sum of exponential decays. The QWD of each sample was extracted from the bleach signals before the exciton transfer process (~0.7 ps) and quantified using the following equation^50^:

1 $$\rho _{n_i} = \frac{{{\int}_{n_i} {\Delta A\;{\mathrm{d}}E} }}{{{\int}_n {\Delta A\;{\mathrm{d}}E} }}.$$

### First-principle calculation

The crystal structure optimizations of CsPbBr_3_ and CsPbClBr_2_ were calculated by the Vienna ab initio simulation package and the projector-augmented wave method. The plane wave cut-off energy was 500 eV. The geometry was relaxed until all atomic forces were <0.01 eV Å^−1^. The Perdew–Burke–Ernzerhof revised for solids functional exchange–correlation functional was applied for obtaining more precise density functional theory energetics. A Γ-centered 6 × 6 × 6 *k*-point mesh was used for simulation.

### Device fabrication and characterization

ITO-coated glass substrates were cleaned with deionized water, acetone, ethanol, and isopropanol in an ultrasonic bath. The dried substrates were treated under oxygen plasma etching for 2 min. The PEDOT:PSS layers were first spin-coated onto the substrates at 4000 r.p.m. for 60 s and annealed at 150 °C for 15 min in air. After cooling, samples were transferred into a nitrogen-filled glove box (O_2_, <0.1 p.p.m., H_2_O, <0.1 p.p.m.). The TFB layers were deposited by spin-coating its chlorobenzene solution (8 mg ml^−1^) at 2000 r.p.m. for 45 s, followed by annealing at 150 °C for 30 min. Then oxygen plasma was used again to treat the TFB films for 2 min. The perovskite films were deposited in the glovebox by spin-coating the precursor solution at 4000 r.p.m. for 90 s. Ethyl acetate, used as anti-solvent, was dropped at ~18 s after the beginning of rotation. Finally, all samples were transferred to a thermal evaporator. TPBi (40 nm), LiF (1 nm), and Al (100 nm) were deposited under a high vacuum of ~7 × 10^−7^ torr. The active area of the devices was 4 mm^2^.

All devices were characterized at room temperature in a nitrogen-filled glovebox, using a Keithley 2400 source meter and a SpectraScan PR-788 spectroradiometer. The EQEs of devices were calculated with the assumption of Lambertian emission pattern of all devices, according to the previously reported method^61,62^.

## Supplementary information

Supplementary Information

Description of Additional Supplementary Files

Supplementary Movie 1

## Data Availability

The data that support the findings of this study are available from the corresponding author upon reasonable request.
