# Supplementary material for: Dimension control of in situ fabricated CsPbClBr2 nanocrystal films toward efficient blue light-emitting diodes
Source: Nat Commun. 2020 Dec 22;11:6428. doi: 10.1038/s41467-020-20163-7 (PMC7755912; doi:10.1038/s41467-020-20163-7)
Supplement: Supplementary file 1 — Supplementary Information [file 41467_2020_20163_MOESM1_ESM.pdf]

SUPPLEMENTARY INFORMATION

Dimension control of *in situ* fabricated $CsPbClBr_2$ nanocrystal films toward efficient blue light-emitting diodes

Wang *et al.*

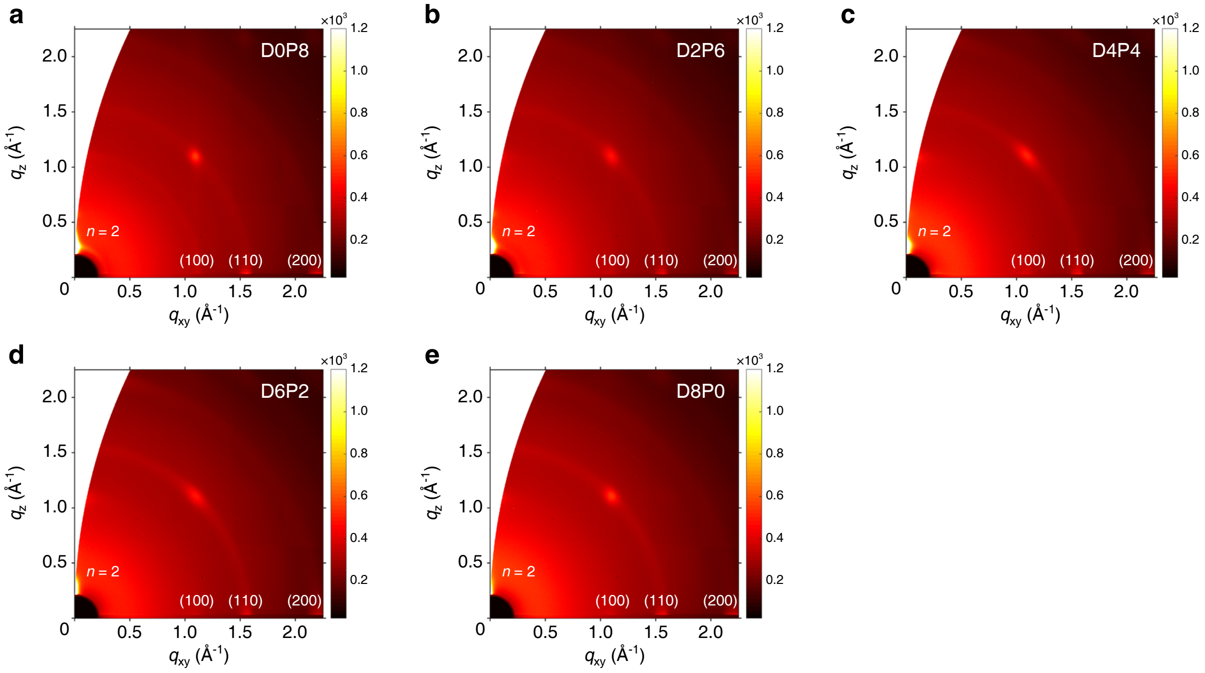

Supplementary Figure 1 | GIWAXS images. a D0P8. **b** D2P6. **c** D4P4. **d** D6P2. **e** D8P0. Taking into account the Ewald sphere, GIWAXS patterns are shown after missing wedge correction.

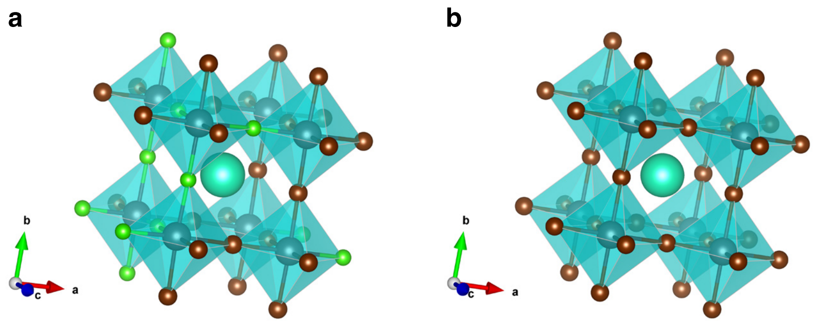

Supplementary Figure 2 | The relaxed crystal structures. a $CsPbClBr_2$. **b** $CsPbBr_3$. The cyan, gray, green and brown balls represent the atoms of Cs, Pb, Cl and Br, respectively.

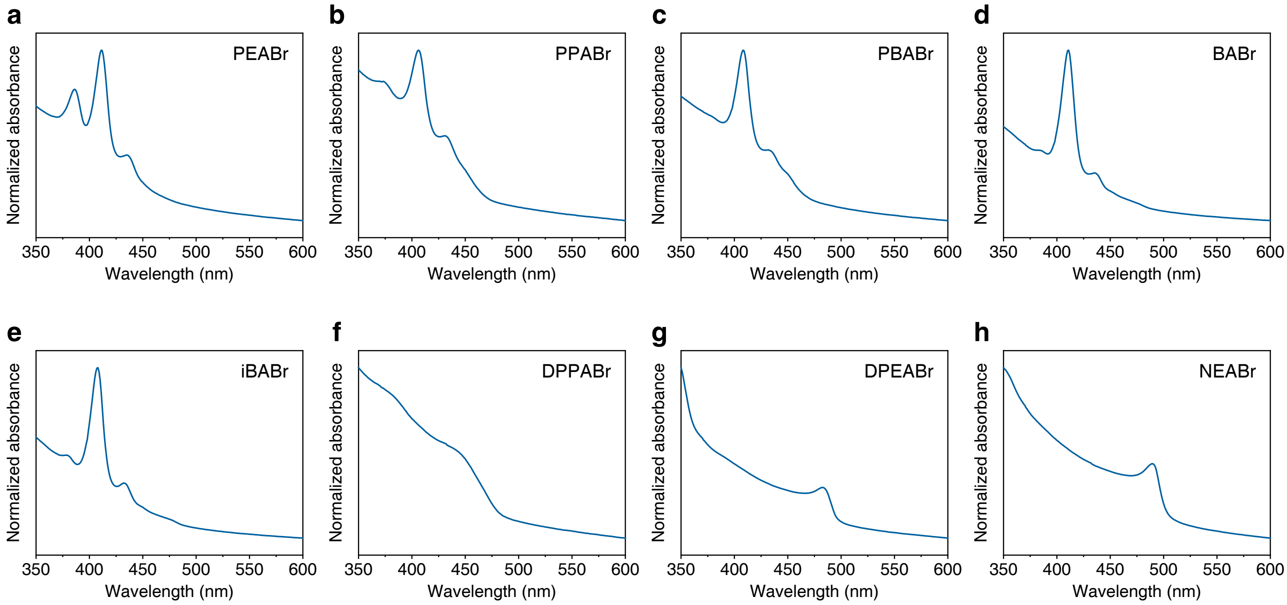

Supplementary Figure 3 | Normalized absorption spectra of $CsPbClBr_2$ nanocrystal films with single ligand. The films using PEABr (**a**), PPABr (**b**), PBABr (**c**), BABr (**d**) and iBABr (**e**) show featured absorption peaks of small-*n* domains (n = 1, 2, 3). In comparison, these peaks are not distinguishable for the films using DPPABr (**f**), DPEABr (**g**) and NEABr (**h**), suggesting the formation of large-*n* domains ($n > 3$).

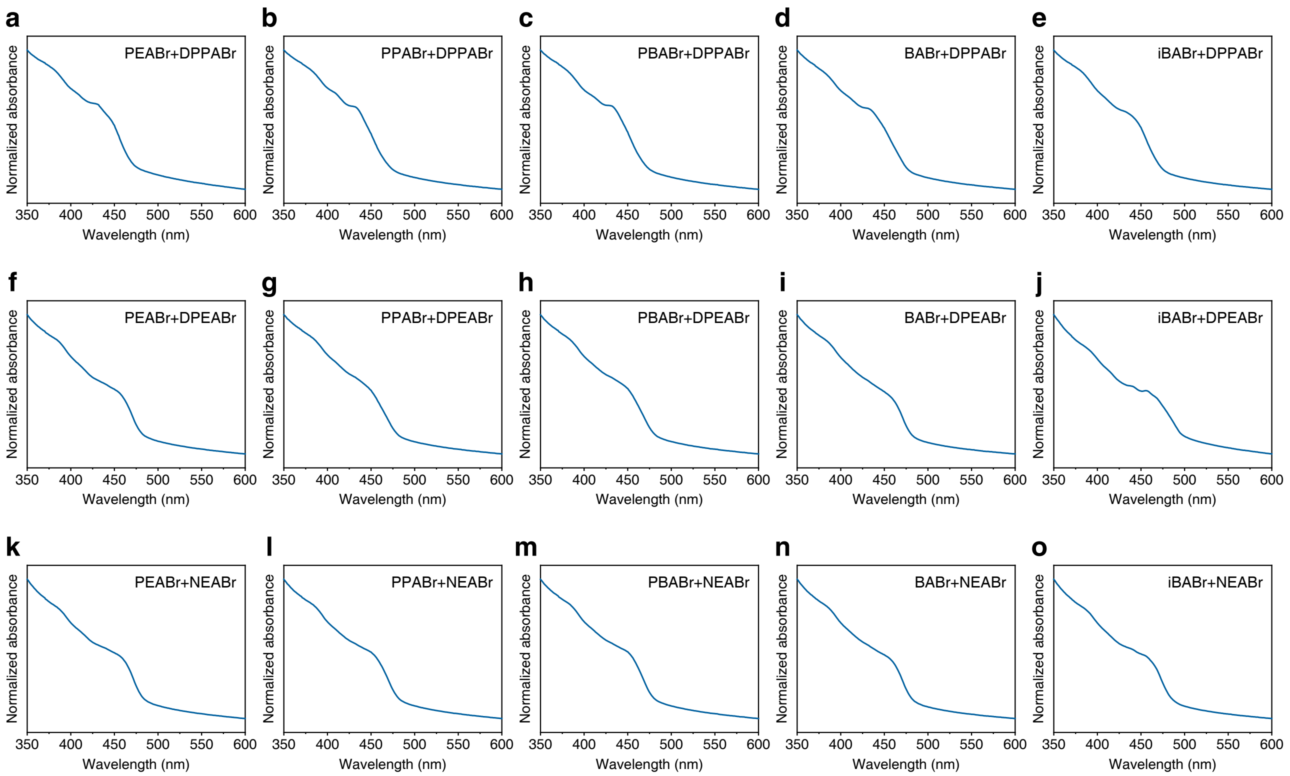

Supplementary Figure 4 | Normalized absorption spectra of $CsPbClBr_2$ nanocrystal films with dual-ligand (a–o). All the films were obtained using a mixture of the two types of ligands with a molar ratio of 1:1.

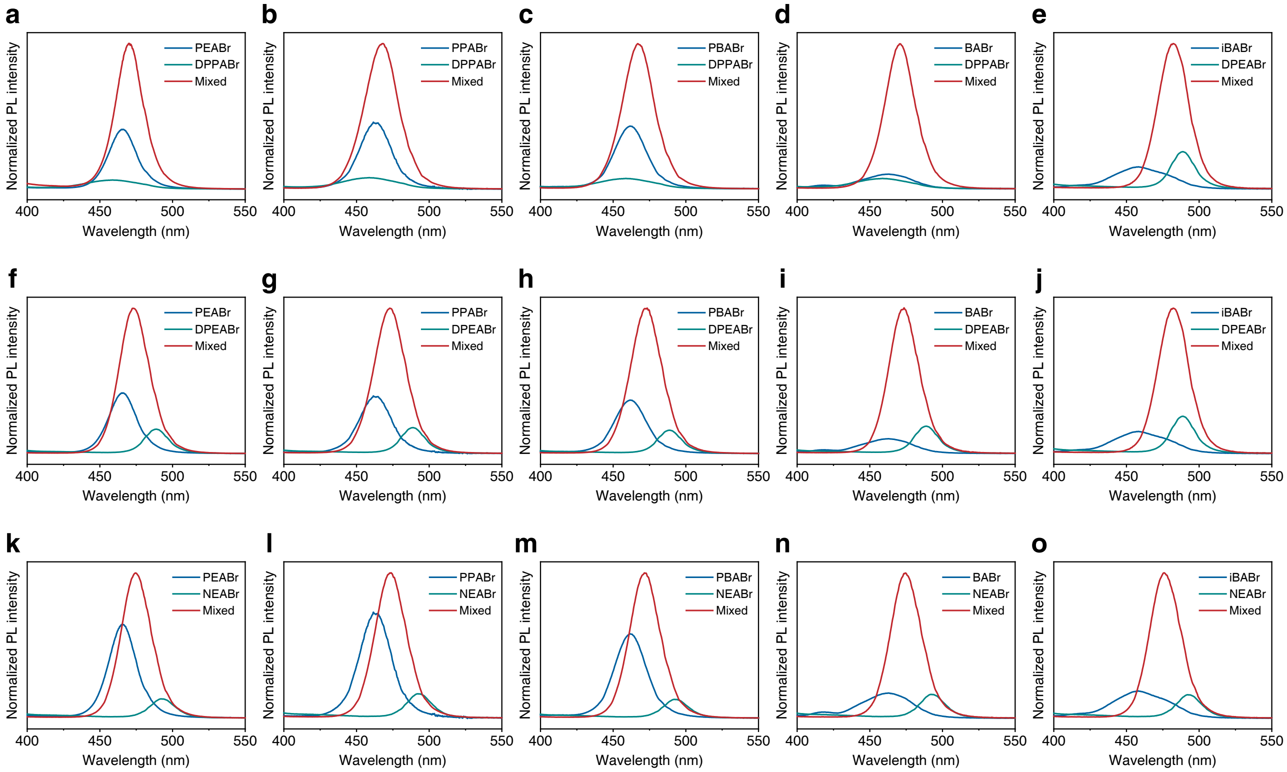

Supplementary Figure 5 | Normalized PL spectra of $CsPbClBr_2$ nanocrystal films obtained from single ligand and dual-ligand (a–o). All the films using dual-ligand show significant PL enhancement in comparison with the samples using single ligand.

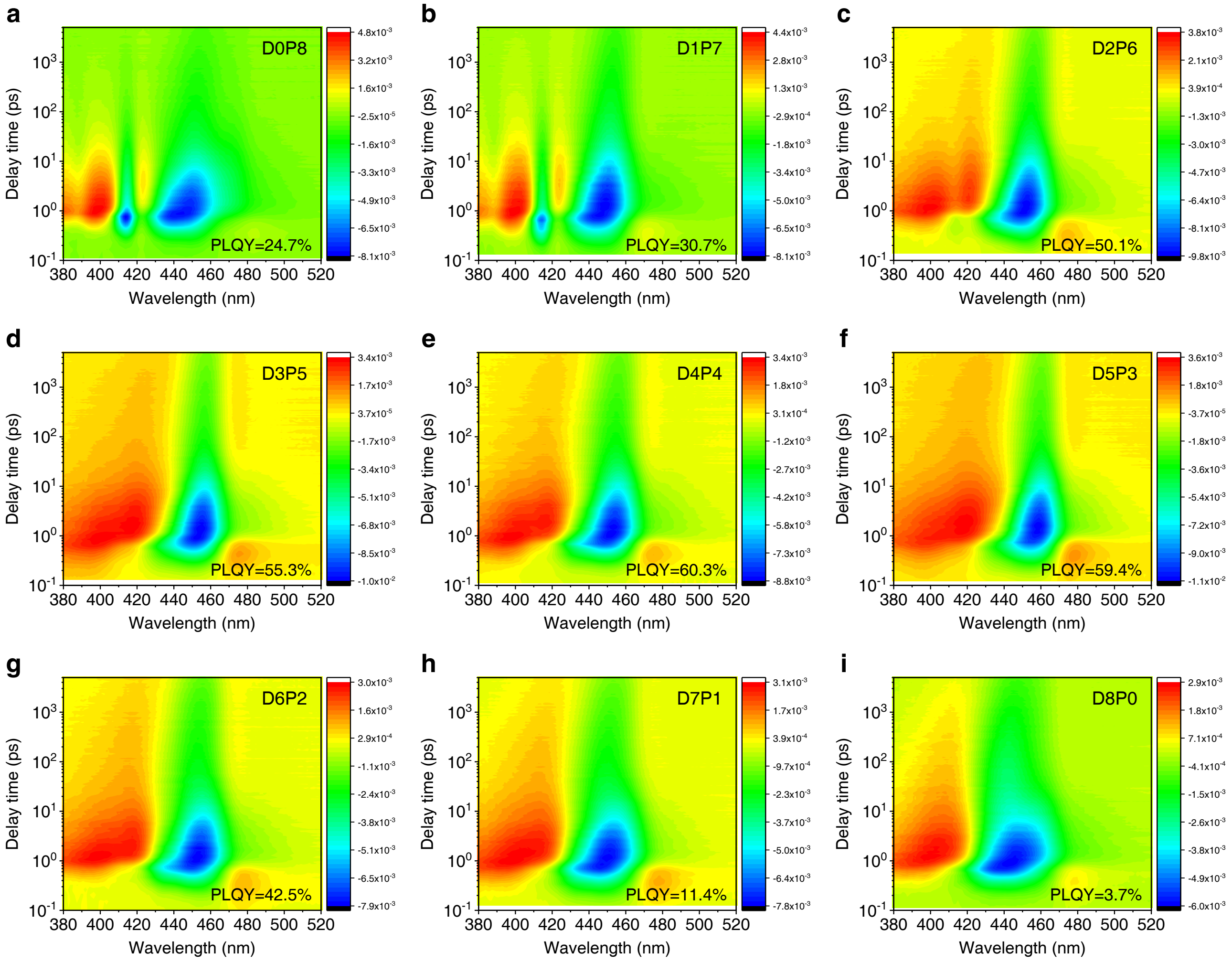

Supplementary Figure 6 | TA color maps. a D0P8. **b** D1P7. **c** D2P6. **d** D3P5. **e** D4P4. **f** D5P3. **g** D6P2. **h** D7P1. **i** D8P0. D0P8 and D1P7 shows two distinctive bleach peaks of small-*n* domains ($n = 1$ and $n = 2$). As the proportion of DPPABr increases, the bleach peaks of small-*n* domains disappear, and the TA map shows a single broad bleach peak (425–470 nm). The FWHMs of such bleach peaks first reduce for the samples of D3P5, D4P4 and D5P3, implying narrow QWDs. As the proportion of DPPABr continues to increase, the FWHMs of such bleach peaks start to increase for the samples of D6P2, D7P1 and D8P0, implying broad QWDs. The PLQYs of these samples are marked on the TA maps respectively.

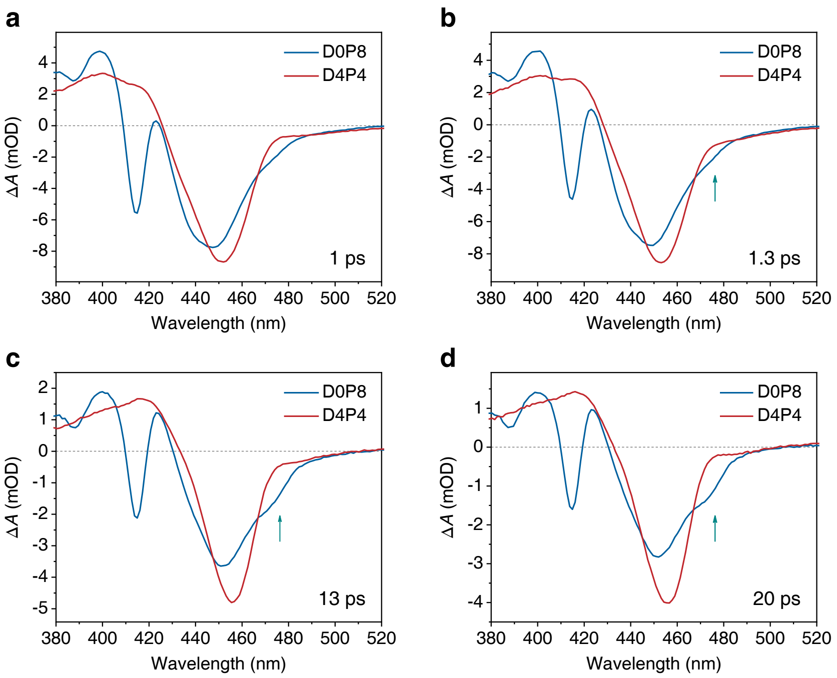

Supplementary Figure 7 | Selected TA spectra in the delay time range of 1–20 ps for the samples of D0P8 and D4P4. a 1 ps. **b** 1.3 ps. **c** 13 ps. **d** 20 ps. As shown in Fig. 4a, similar evolution of bleach peaks are observed in D0P8 and D4P4. However, the bleach signals corresponding to the large-*n* domains are observed after 1 ps in D0P8 (see the arrows), which results in distinct differences on the FWHM of the bleach peaks in these two samples, as shown in Fig. 4b.

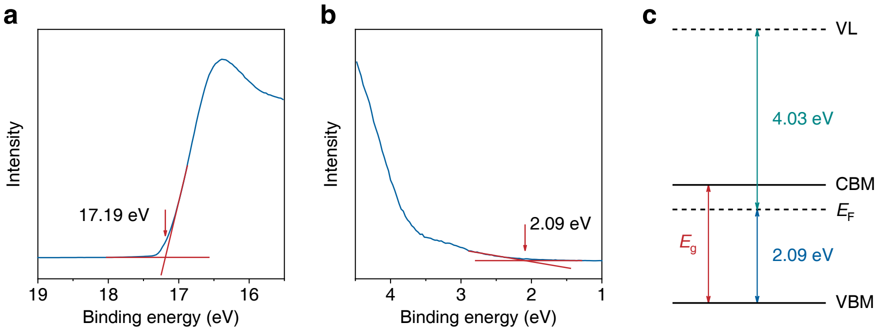

Supplementary Figure 8 | Energy level of D4P4. a, **b** Zoomed-in UPS spectra of the cut-off region (**a**) and band-edge region (**b**). **c** The energy level diagram. The Fermi energy (E_F) is -4.03 eV, which is obtained by calculating the work function (ϕ) through $\phi = hv\text{-}E_{\text{cut-off}}$, where hv is the incident photon energy (hv = 21.22 eV as HeI source is used here) and $E_{\text{cut-off}}$ is the measured secondary-cut-off energy. The lowest unoccupied molecular orbital (LUMO) and highest occupied molecular orbital (HOMO) of D4P4 are determined by the optical bandgap and the onset of photoelectron intensity with respect to E_F, which are -3.48 eV and -6.12 eV, respectively.

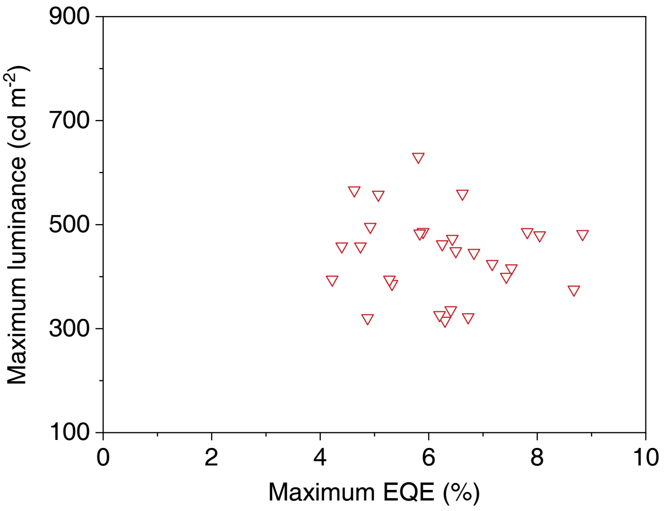

Supplementary Figure 9 | Statistical data of maximum EQE and maximum luminance from 28 devices (D4P4). An average EQE of 6.2% and an average luminance of 442 cd m^{-2} were obtained.

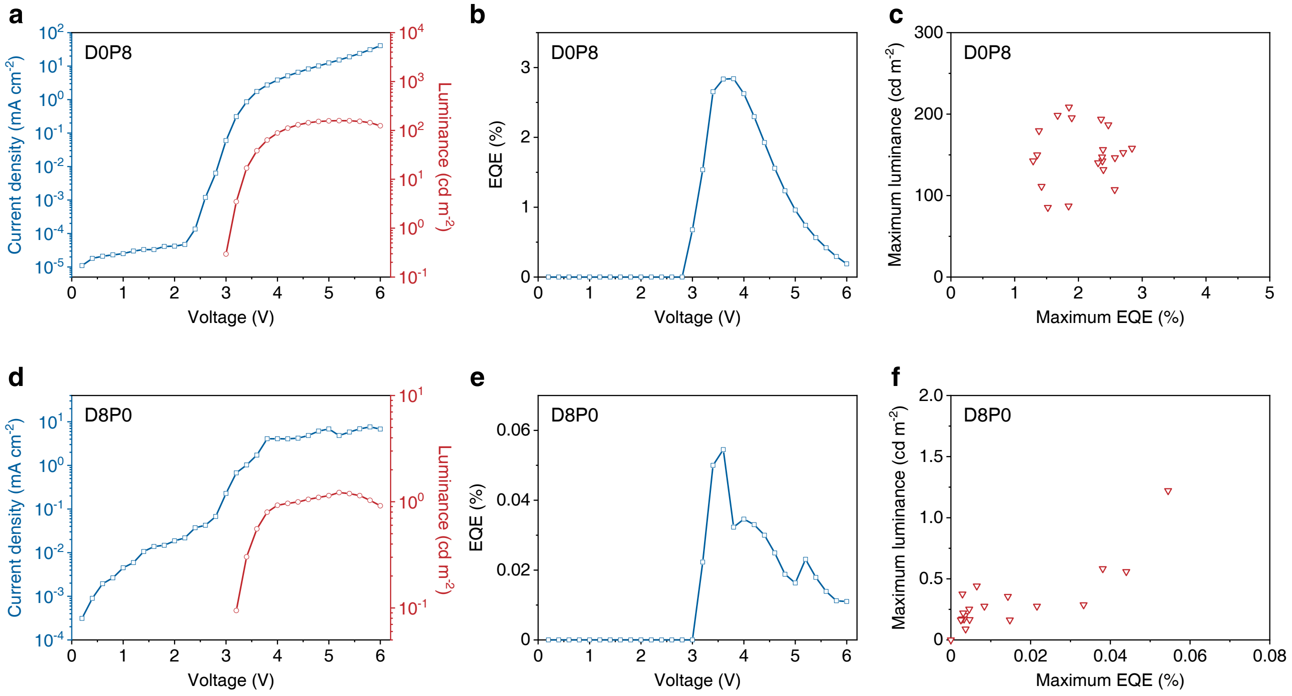

Supplementary Figure 10 | Device performance of D0P8 and D8P0. a, **b** Current density–luminance–voltage (**a**) and EQE–voltage (**b**) characteristics of the best-performance device of D0P8. A maximum EQE of 2.8% and a maximum luminance of 209 cd m^{-2} were obtained. **c** Statistical data of maximum EQE and maximum luminance from 20 devices (D0P8). The statistical data show an average EQE of 2.1% and an average luminance of 151 cd m^{-2}. **d**, **e** Current density–luminance–voltage (**d**) and EQE–voltage (**e**) characteristics of the best-performance device of D8P0. A maximum EQE of 0.05% and a maximum luminance of 1.2 cd m^{-2} were obtained. **f** Statistical data of maximum EQE and maximum luminance from 20 devices (D8P0). The statistical data show an average EQE of 0.01% and an average luminance of 0.3 cd m^{-2}.

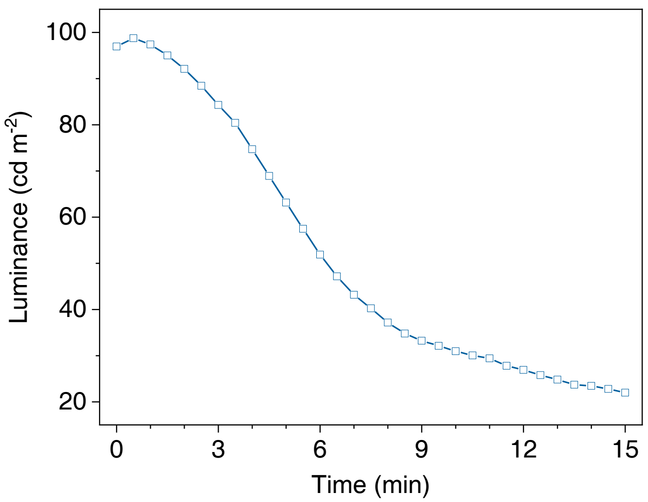

Supplementary Figure 11 | The operational lifetime of the blue PeLED. The device was operating at a constant current density of 1.95 mA cm^{-2} with an initial luminance around 100 cd m^{-2}. The T_{50} defined as the time when luminance drops to 50% of its initial value is 6.3 min.

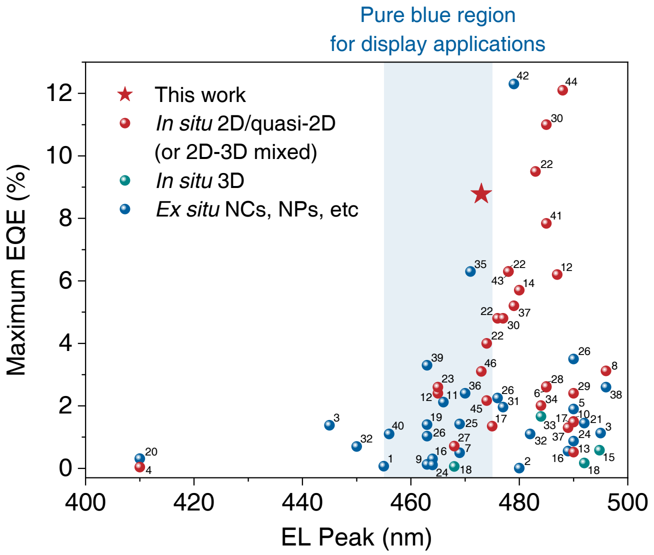

Supplementary Figure 12 | Comparison of our device and other blue PeLEDs with EL peak in the range of 400–500 nm. A record EQE of 8.8 % is achieved in the pure blue region (455–475 nm) in this work.

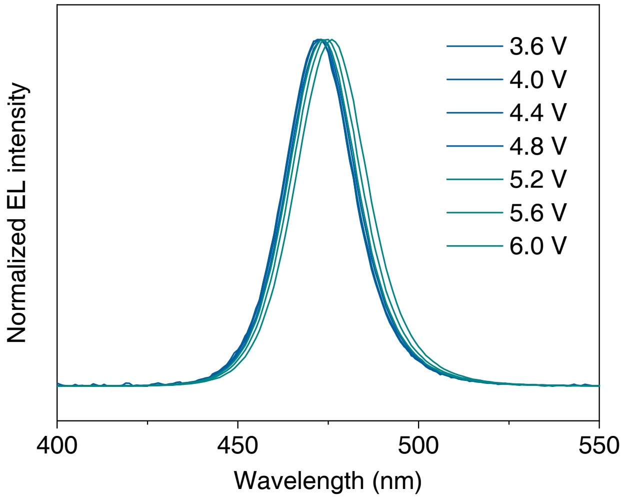

Supplementary Figure 13 | Normalized EL spectra of D4P4 under different applied voltages.

The EL peak is located at ~473 nm before 5.2 V. During the operation from 5.2 V to 6.0 V, the EL peak gradually shifts from 473 nm to 476 nm.

Supplementary Table 1 | The theoretical crystal plane spacing of $CsPbClBr_2$ and $CsPbBr_3$. The lattice constant *d* (Å) of cubic 3D $CsPbClBr_2$ perovskite is obtained by first-principle calculation. As a verification, the lattice constant of cubic 3D $CsPbBr_3$ is calculated using the same method, which is consistent with the standard PDF card (PDF#18-0364). Through the calculation of $q = 2\pi/d$, three peaks at 1.09 $Å^{-1}$, 1.55 $Å^{-1}$, 2.18 $Å^{-1}$ identified in the GIWAXS pattern (Fig. 1b and Supplementary Fig. 1) can be assigned to the (100), (110), (200) crystal planes of 3D $CsPbClBr_2$ nanocrystals.

Crystal plane	*d* (Å) $CsPbClBr_2$	*d* (Å) $CsPbBr_3$
(100)	5.76	5.86
(110)	4.07	4.14
(200)	2.88	2.93
(201)	2.58	2.62
(211)	2.35	2.39
(202)	2.04	2.07

Supplementary Table 2 | Fitting parameters of the TA kinetics. The kinetics are fit by a sum of convoluted exponentials: $S(t) \propto a_1 \cdot e^{-t/\tau_1} + a_2 \cdot e^{-t/\tau_2} + a_3 \cdot e^{-t/\tau_3}$, where a_1, a_2, a_3 are the amplitudes and τ_1, τ_2, τ_3 are the decay time constants.

		τ_1 /ps (a_1)	τ_2 /ps (a_2)	τ_3 /ps (a_3)
D0P8	414 nm	0.37±0.03 (76.5%)	13.4±1.8 (17.2%)	253.5±59.9 (6.3%)
	435 nm	0.55±0.08 (55.0%)	4.4±0.5 (37.9%)	331.8±61.4 (7.1%)
	450 nm	6.9±0.4 (65.4%)	78.2±13.5 (19.2%)	4226±584 (15.4%)
	461 nm	11.1±0.6 (65.6%)	85.9±17.5 (16.7%)	6778±893 (17.7%)
D4P4	435 nm	0.30±0.09 (72.9%)	1.7±0.5 (24.6%)	294.3±172.0 (2.5%)
	452 nm	7.6±0.5 (60.5%)	75.8±18.6 (16.9%)	7632±1180 (22.6%)
	462 nm	13.5±0.8 (65.7%)	133.7±40.0 (13.0%)	14150±3660 (21.3%)
D8P0	435 nm	1.7±0.1 (59.7%)	86.1±17.1 (17.6%)	3107±401 (22.7%)
	447 nm	5.4±0.3 (56.5%)	82.6±16.4 (16.5%)	6868±787 (27.0%)
	460 nm	9.6±1.0 (63.8%)	59.7±19.6 (19.6%)	10900±3410 (16.6%)

Supplementary References

1. Song, J. et al. Quantum dot light-emitting diodes based on inorganic perovskite cesium lead halides ($CsPbX_3$). *Adv. Mater.* **27**, 7162–7167 (2015).
2. Li, G. et al. Highly efficient perovskite nanocrystal light-emitting diodes enabled by a universal crosslinking method. *Adv. Mater.* **28**, 3528–3534 (2016).
3. Deng, W. et al. Organometal halide perovskite quantum dot light-emitting diodes. *Adv. Funct. Mater.* **26**, 4797–4802 (2016).
4. Liang, D. et al. Color-pure violet-light-emitting diodes based on layered lead halide perovskite nanoplates. *ACS Nano* **10**, 6897–6904 (2016).
5. Pan, J. et al. Highly efficient perovskite-quantum-dot light-emitting diodes by surface engineering. *Adv. Mater.* **28**, 8718–8725 (2016).
6. Wang, Q., Ren, J., Peng, X., Ji, X. & Yang, X. Efficient sky-blue perovskite light-emitting devices based on ethylammonium bromide induced layered perovskites. *ACS Appl. Mater. Interfaces* **9**, 29901–29906 (2017).
7. Gangishetty, M. K., Hou, S., Quan, Q. & Congreve, D. N. Reducing architecture limitations for efficient blue perovskite light-emitting diodes. *Adv. Mater.* **30**, 1706226 (2018).
8. Shang, Y., Li, G., Liu, W. & Ning, Z. Quasi-2D inorganic $CsPbBr_3$ perovskite for efficient and stable light-emitting diodes. *Adv. Funct. Mater.* **28**, 1801193 (2018).
9. Wu, Y. et al. In situ passivation of $PbBr_6^{4-}$ octahedra toward blue luminescent $CsPbBr_3$ nanoplatelets with near 100% absolute quantum yield. *ACS Energy Lett.* **3**, 2030–2037 (2018).

10. Xing, J. et al. Color-stable highly luminescent sky-blue perovskite light-emitting diodes. *Nat. Commun.* **9**, 3541 (2018).

11. Hou, S., Gangishetty, M. K., Quan, Q. & Congreve, D. N. Efficient blue and white perovskite light-emitting diodes via manganese doping. *Joule* **2**, 1–13 (2018).

12. Vashishtha, P., Ng, M., Shivarudraiah, S. B. & Halpert, J. E. High efficiency blue and green light-emitting diodes using Ruddlesden–Popper inorganic mixed halide perovskites with butylammonium interlayers. *Chem. Mater.* **31**, 83–89 (2019).

13. Wang, K. et al. Efficient and color-tunable quasi-2D $CsPbBr_xCl_{3-x}$ perovskite blue light-emitting diodes. *ACS Photonics* **6**, 667–676 (2019).

14. Li, Z. et al. Modulation of recombination zone position for quasi-two-dimensional blue perovskite light-emitting diodes with efficiency exceeding 5%. *Nat. Commun.* **10**, 1027 (2019).

15. Gangishetty, M. K., Sanders, S. N. & Congreve, D. N. Mn^{2+} doping enhances the brightness, efficiency, and stability of bulk perovskite light-emitting diodes. *ACS Photonics* **6**, 1111–1117 (2019).

16. Hoye, R. L. Z. et al. Identifying and reducing interfacial losses to enhance color-pure electroluminescence in blue-emitting perovskite nanoplatelet light-emitting diodes. *ACS Energy Lett.* **4**, 1181–1188 (2019).

17. Jiang, Y. et al. Spectra stable blue perovskite light-emitting diodes. *Nat. Commun.* **10**, 1868 (2019).

18. Wang, H., Zhao, X., Zhang, B. & Xie, Z. Blue perovskite light-emitting diodes based on RbX-doped polycrystalline $CsPbBr_3$ perovskite films. *J. Mater. Chem. C* **7**, 5596–5603 (2019).

19. Ochsenbein, S. T., Krieg, F., Shynkarenko, Y., Rainò, G. & Kovalenko, M. V. Engineering color-stable blue light-emitting diodes with lead halide perovskite nanocrystals. *ACS Appl. Mater. Interfaces* **11**, 21655–21660 (2019).

20. Deng, W. et al. 2D Ruddlesden–Popper perovskite nanoplate based deep-blue light-emitting diodes for light communication. *Adv. Funct. Mater.* **29**, 1903861 (2019).

21. Ren, Z. et al. Hole transport bilayer structure for quasi-2D perovskite based blue light-emitting diodes with high brightness and good spectral stability. *Adv. Funct. Mater.* **29**, 1905339 (2019).

22. Liu, Y. et al. Efficient blue light-emitting diodes based on quantum-confined bromide perovskite nanostructures. *Nat. Photonics* **13**, 760–764 (2019).

23. Yuan S. et al. Optimization of low-dimensional components of quasi-2D perovskite films for deep-blue light-emitting diodes. *Adv. Mater.* **31**, 1904319 (2019).

24. Todorović, P. et al. Spectrally tunable and stable electroluminescence enabled by rubidium doping of $CsPbBr_3$ nanocrystals. *Adv. Optical Mater.* **7**, 1901440 (2019).

25. Zhang, C. et al. Surface ligand engineering toward brightly luminescent and stable cesium lead halide perovskite nanoplatelets for efficient blue-light-emitting diodes. *J. Phys. Chem. C* **123**, 26161–26169 (2019).

26. Shynkarenko, Y. et al. Direct synthesis of quaternary alkylammonium-capped perovskite nanocrystals for efficient blue and green light-emitting diodes. *ACS Energy Lett.* **4**, 2703–2711 (2019).

27. Tan, Z. et al. Spectrally stable ultra-pure blue perovskite light-emitting diodes boosted by square-wave alternating voltage. *Adv. Optical Mater.* **8**, 1901094 (2020).

28. Jin, Y. et al. Synergistic effect of dual ligands on stable blue quasi-2D perovskite light-emitting diodes. *Adv. Funct. Mater.* **30**, 1908339 (2020).

29. Zeng, S., Shi, S., Wang, S. & Xiao, Y. Mixed-ligand engineering of quasi-2D perovskites for efficient sky-blue light-emitting diodes. *J. Mater. Chem. C* **8**, 1319–1325 (2020).

30. Wang, Q. et al. Efficient sky-blue perovskite light-emitting diodes via photoluminescence enhancement. *Nat. Commun.* **10**, 5633 (2019).

31. Yang, F. et al. Efficient and spectrally stable blue perovskite light-emitting diodes based on potassium passivated nanocrystals. *Adv. Funct. Mater.* **30**, 1908760 (2020).

32. Chen, H. et al. Structural and spectral dynamics of single-crystalline Ruddlesden–Popper phase halide perovskite blue light-emitting diodes. *Sci. Adv.* **6**, eaay4045 (2020).

33. Wang, H. et al. Bright and color-stable blue-light-emitting diodes based on three-dimensional perovskite polycrystalline films via morphology and interface engineering. *J. Phys. Chem. Lett.* **11**, 1411–1418 (2020).

34. Yuan, F. et al. A cocktail of multiple cations in inorganic halide perovskite toward efficient and highly stable blue light-emitting diodes. *ACS Energy Lett.* **5**, 1062–1069 (2020).

35. Zheng, X. et al. Chlorine vacancy passivation in mixed halide perovskite quantum dots by organic pseudohalides enables efficient Rec. 2020 blue light-emitting diodes. *ACS Energy Lett.* **5**, 793–798 (2020).

36. Pan, G. et al. Bright blue light emission of Ni^{2+} ion-doped $CsPbCl_xBr_{3-x}$ perovskite quantum dots enabling efficient light-emitting devices. *ACS Appl. Mater. Interfaces* **12**, 14195–14202 (2020).

37. Ma, D. et al. Chloride insertion–immobilization enables bright, narrowband, and stable blue-emitting perovskite diodes. *J. Am. Chem. Soc.* **142**, 5126–5134 (2020).

38. Ye, F. et al. Spectral tuning of efficient $CsPbBr_xCl_{3-x}$ blue light-emitting diodes via halogen exchange triggered by benzenesulfonates. *Chem. Mater.* **32**, 3211–3218 (2020).

39. Yao, J. et al. Calcium-tributylphosphine oxide passivation enables the efficiency of pure-blue perovskite light-emitting diode up to 3.3%. *Sci. Bull.* **65**, 1150–1153 (2020).

40. Chiba, T. et al. Blue perovskite nanocrystal light-emitting devices via the ligand exchange with adamantane diamine. *Adv. Optical Mater.* **8**, 2000289 (2020).

41. Wang, F. et al. High performance quasi-2D perovskite sky-blue light-emitting diodes using a dual-ligand strategy. *Small* **16**, 2002940 (2020).

42. Dong, Y. et al. Bipolar-shell resurfacing for blue LEDs based on strongly confined perovskite quantum dots. *Nat. Nanotechnol.* **15**, 668–674 (2020).

43. Wang, Y. et al. Chelating-agent-assisted control of $CsPbBr_3$ quantum well growth enables stable blue perovskite emitters. *Nat. Commun.* **11**, 3674 (2020).

44. Chu, Z. et al. Large cation ethylammonium incorporated perovskite for efficient and spectra stable blue light-emitting diodes. *Nat. Commun.* **11**, 4165 (2020).

45. Worku, M. et al. Phase control and in situ passivation of quasi-2D metal halide perovskites for spectrally stable blue light-emitting diodes. *ACS Appl. Mater. Interfaces* **12**, 45056–45063 (2020).

46. Zhang, F. et al. Chlorine distribution management for spectrally stable and efficient perovskite blue light-emitting diodes. *Nano Energy* **79**, 105486 (2021).
