# Supplementary material for: Dimension control of in situ fabricated CsPbClBr2 nanocrystal films toward efficient blue light-emitting diodes
Source: Nat Commun. 2020 Dec 22;11:6428. doi: 10.1038/s41467-020-20163-7 (PMC7755912; doi:10.1038/s41467-020-20163-7)
Supplement: Supplementary file 2 — Description of Additional Supplementary Files [file 41467_2020_20163_MOESM2_ESM.pdf]

Description of Additional Supplementary Files

File Name: Supplementary Movie 1
Description: *In situ* fabrication of $CsPbClBr_2$ nanocrystal film (D4P4)
